# Cell Type Influences Local Delivery of Biomolecules from a Bioinspired Apatite Drug Delivery System

**DOI:** 10.3390/ma11091703

**Published:** 2018-09-13

**Authors:** Jumana Alhamdi, Emily Jacobs, Gloria Gronowicz, Nadia Benkirane-Jessel, Marja Hurley, Liisa Kuhn

**Affiliations:** 1 Department of Biomedical Engineering, University of Connecticut Health Center, Farmington, CT 06030, USA; alhamdijumana@gmail.com (J.A.); emily.e.jacobs@gmail.com (E.J.); 2 Department of Surgery, University of Connecticut Health, Farmington, CT 06030, USA; gronowicz@uchc.edu; 3 French National Institute of Health and Medical Research (INSERM), UMR 1260, Faculté de Médecine, University of Strasbourg, 67085 Strasbourg, France; nadia.jessel@inserm.fr; 4 Department of Medicine, University of Connecticut Health, Farmington, CT 06030, USA; hurley@uchc.edu

**Keywords:** biomimetic calcium phosphate, bone like apatite, polyelectrolyte multilayers, drug delivery, cell-biomaterial interactions

## Abstract

Recently, the benefit of step-wise sequential delivery of fibroblast growth factor-2 (FGF-2) and bone morphogenetic protein-2 from a bioinspired apatite drug delivery system on mouse calvarial bone repair was demonstrated. The thicknesses of the nanostructured poly-l-Lysine/poly-l-Glutamic acid polyelectrolyte multilayer (PEM) and the bone-like apatite barrier layer that make up the delivery system, were varied. The effects of the structural variations of the coating on the kinetics of cell access to a cytotoxic factor delivered by the layered structure were evaluated. FGF-2 was adsorbed into the outer PEM, and cytotoxic antimycin-A (AntiA) was adsorbed to the substrate below the barrier layer to detect the timing of the cell access. While MC3T3-E1 osteoprogenitor cells accessed AntiA after three days, the RAW 264.7 macrophage access occurred within 4 h, unless the PEM layer was removed, in which case the results were reversed. Pits were created in the coating by the RAW 264.7 macrophages and initiated delivery, while the osteoprogenitor cell access to drugs occurred through a solution-mediated coating dissolution, at junctions between the islands of crystals. Macrophage-mediated degradation is therefore a mechanism that controls drug release from coatings containing bioinspired apatite.

## 1. Introduction

Bone is a unique organ that is in a state of constant remodeling, in order to maintain a strong structure capable of supporting the varied forces applied to it. Injury initiates the site-specific processes of resorption and formation, which occur by the coupled actions of osteoblasts, osteocytes, osteoclasts, bone lining cells, and macrophages [1,2,3]. The recruitment, proliferation, and differentiation of cells participating in bone injury repair and remodeling are controlled by multiple growth factors; such as, transforming growth factor beta 1 (TGFβ1) [4,5], insulin-like growth factor 1 (IGF-1) [6,7], bone morphogenetic protein-2 (BMP-2) [8], and fibroblast growth factor-2 (FGF-2) [9,10,11,12]. Some of these factors are produced and released from the cells participating in the repair, while others become entrapped within the extracellular matrix and are re-released by the actions of bone matrix resorbing cells like osteoclasts and macrophages. 

Implantable biomaterial delivery systems have been developed, which mimic the delivery of multiple factors to guide the tissue regeneration process [13,14,15,16,17,18]. Systems of particular value are those that deliver the molecules in a highly localized manner, because that reduces the off target effects of the potent molecules being delivered [19]. The combined use of a biomimetic calcium phosphate (bCaP) barrier layer with a poly-l-Lysine (PLLys) and poly-l-Glutamic acid (PLGlut) polyelectrolyte multilayer (PEM) is one of the few systems capable of non-overlapping and the sequential delivery of two factors [20,21]. PEM coatings are generated by layer-by-layer assembly involving deposition of the polyelectrolyte molecules on a surface by many different techniques, including dipping into alternate solutions of differently charged molecules [17,22,23]. PEM coatings and the delivery of biomolecules from PEM can be tuned by varying the dipping solution pH, temperature, ionic strength, and the molecules that make up the PEM coatings [24,25,26,27]. Polyelectrolyte multilayers provide a non-denaturing reservoir for growth factors [28,29,30]. The adsorption of biomolecules into a layered structure like PEM/bCaP with spacing between the various molecules mimics biomolecule entrapment in the extracellular matrix that occurs during bone formation. 

Despite the fact that no growth factor release was detected in the release study supernatants from the bCaP/PEM system by sensitive methods like enzyme-linked immunosorbent assay (ELISA), the coating could influence the proliferation and viability of the cells in direct contact with the coating [21]. The advantage of the highly localized, step-wise delivery of FGF-2 and BMP-2 from this bioinspired delivery system on in vivo young mouse calvarial bone repair was demonstrated [20], which validated the highly localized delivery and low dose approach. The biological impact of the structure and the composition of materials used in the PEM/bCaP nanostructure on the biomolecule delivery profile were unknown, and thus investigated by the present study.

Monocytes/macrophages are one of the first cell types that interact with implanted biomaterials [31,32,33]; therefore, to better understand the localized effect of the bCaP/PEM nanostructure on the cells that come in contact with the coating after implantation, the present study included murine-derived RAW 264.7 macrophages, in addition to MC3T3-E1 osteoprogenitors, for the cell culture bioassays. The use of these two cells types recapitulates the cells present in the early inflammatory and bone remodeling events, which mediate the degradation of the extracellular matrix adjacent to the injury and release factors themselves. 

The constructive use of cell-mediated degradation has led others to include matrix metalloprotease (MMP) sensitive ligands within hydrogels, in order to increase the growth factor release [34]. In related studies, the resorptive activities of osteoclasts were shown to be associated with an in vivo release of BMP-2 embedded within the calcium phosphate coatings [35,36]. Given the involvement of cells with the degradation process, the extent of the cell-mediated delivery is another important aspect to study, in addition to the standard release studies involving diffusion out into physiological solutions. Therefore, the extent to which the biomaterial parameters of the bCaP/PEM system; such as, the number of layers of PEM, structure of PEM molecules, and thickness of bCaP, altered the kinetics of the cell access to the factors embedded in the bCaP/PEM coating were investigated in the present studies.

## 2. Materials and Methods

### 2.1. Materials Fabrication

**Figure 1:**
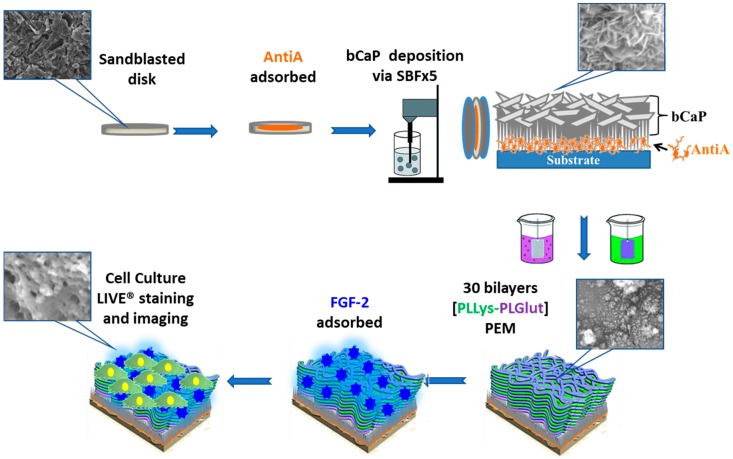
Schematic representation of the bone-like apatite coating (bCaP)/Poly-d-Glutamic Acid (PEM-D) application process and analysis techniques.

A schematic of the method for producing the drug delivery coating evaluated in the studies is shown in Figure 1.

#### 2.1.1. bCaP Application with or without Antimycin A

The bCaP deposition procedure followed a previously reported method [21,37]. All of the reagents were used as received from Sigma-Aldrich. Briefly, ultraviolet light-sterilized, aluminum oxide sandblasted tissue culture polystyrene disks (TCPsb) (NUNC, Rochester, NY, USA), which were 22 mm in diameter, were coated, as previously described, with bCaP mineral crystals via extended immersion in two different highly (5×) concentrated simulated body fluid (SBF) solutions described in full detail in the literature [21,37,38]. The components of the two solutions are the ions present in body fluid, such as calcium, sodium, magnesium, phosphorus, chloride, and so on. The first solution, Solution A, contains more inhibitors for calcium phosphate crystallization, thereby forcing an amorphous structure to be deposited initially on the substrate, which serves as a base layer for the crystalline carbonated apatite that grows when exposed to the second solution, Solution B.

The relationship between the bCaP thickness and immersion time in the two solutions was reported previously [38,39,40]. For these studies, the length of time in the second solution (Solution B) was increased from 7 to 24 h, to form a thicker coating. As the bCaP thickness reaches an equilibrium when the ions are depleted below the growth threshold, which depends on the temperature and other ions present, a group of samples kept for 24 h in Solution B were immersed again in another freshly made Solution B for an additional 24 h to create an even thicker layer. Those samples were denoted as 48 h. After removal from Solution B, all of the samples were sonicated briefly, rinsed with distilled water, and then dehydrated in a series of graded ethanol prior to cell culture studies.

In the studies with the cytotoxic compound Antimycin-A (AntiA), ultraviolet light-sterilized, aluminum oxide sandblasted tissue culture polystyrene disks (TCPsb) (NUNC, Rochester, NY, USA), which were 22 mm in diameter, were covered, on one side only, with 10 μL of 40 mM Antimycin-A (AntiA, Sigma, St. Louis, MO, USA) dissolved in ethanol (for a total of 213 μg/disk), prior to the bCaP coating. This dose was selected based on the cytotoxicity of this dose to MC3T3-E1 determined in our previous studies [21] and comparable to the cytotoxicity of the AntiA to both MC3T3-E1 and RAW 264.7 macrophages [41]. The disks were then rinsed three times in saline and coated with bCaP mineral crystal via extended immersion in two different concentrated SBF solutions, as described above. The side that had received the AntiA coating was marked so that the cells could be applied to the side with the AntiA coating for the bioassays. A 100% binding of the AntiA was assumed based on its poor solubility in water and the hydrophobicity of the drug, as well as previous experiments that showed no cytotoxicity to MC3T3-E1 cells from the rinse solutions [21]. 

Coatings for pulsatile delivery: Another group of coated disks was created by applying two separate 24 h bCaP coatings with separate AntiA doses applied below each one. After the first two step bCaP applications, another 10 μL of 40 mM AntiA dissolved in ethanol was applied onto the first bCaP coating, on the same side coated earlier. The disks were then coated with another layer of bCaP mineral crystals via the two step process, as described earlier. No PEM coating was applied to these samples. The abbreviations for the sample names start with the layer that is furthest away from the surface (e.g., AntiA-bCaP1-AntiA-bCaP2 for the pulsatile delivery structure).

#### 2.1.2. PEM Bilayer Coating

Layer-by-layer PEM was applied by alternate dipping in poly-l-Glutamic acid and poly-l-Lysine solutions with saline rinses between, following a previously reported method [21,42]. Briefly, TCPsb disks coated with bCaP were held vertically in a custom three-dimensional (3D) printed sample holder and automatically dipped into PEM solutions and saline rinses using a histology staining machine (Varistain 24-4, Thermo Shandon, Loughborough, UK). The automatic PEM bi-layer process included an adsorption of poly-l-Glutamic acid (PLGlut) (1 mg/mL, Sigma P4761, St. Louis, MO, USA) for 10 min followed by 1 min in seven saline rinses. This was followed by the adsorption of poly-l-Lysine (PLLys, Sigma P2636, St. Louis, MO, USA) for 10 min, followed by 1 min in seven saline rinses. This cycle was repeated to achieve 30 bilayers (sufficient to completely cover the underlying structure and serve as a reservoir for biomolecules) or 102 bilayers of PEM coating on the disks, which represented the maximum that could be achieved by the automated coating process, despite three complete replacements of the stock PLGlut and PLLys solution. A stock solution replacement was necessary every 30 or so bilayers, due to the carry-over of the electrolyte solutions into the saline rinses by the racks of disks as they were moved in and out of the solutions. Based on previous studies that had shown effective short term tuning of the biological activity of PEM by varying the d/l-enantiomer ratio, due to the reduced ability of the cells to degrade the d-form [43], some of the PEM coatings were made by using Poly-d-Lysine and Poly-d-Glutamic Acid (PEM-D), as compared to the l-form of the polyamino acids, as an alternate means of slowing degradation. 

#### 2.1.3. FGF-2 Factor Absorption

After PEM coating, a carrier free recombinant human FGF-2 (R&D Systems, Minneapolis, MN, USA) in saline was adsorbed to the coated disks. The disks were incubated for 1 h in 0.5 mL of FGF-2 (375 ng/mL) solution, to allow binding to the surface, and were then rinsed three times with saline. Previous studies had determined that the dose of FGF-2 applied this way was 120 ng/disk by enzyme-linked immunosorbent assay (ELISA) on the post-binding and rinse solutions [21].

### 2.2. Characterization

#### 2.2.1. Cell Culture Assays

MC3T3-E1 mouse calvarial osteoprogenitor cells (ATCC, Manassas, VA, USA) or macrophages RAW 264.7 (ATCC, Manassas, VA, USA) were cultured in an Alpha-Minimal Essential Medium (α-MEM, No. 12571, Gibco BRL, Invitrogen), supplemented with 10% fetal bovine serum (FBS), 100 U/mL penicillin, and 100 μg/mL streptomycin sulfate. These two cell types were selected because of their participation in the early stages of bone healing. The cell culture medium was refreshed three times a week until the cells reached 80% confluency, and passages 10–30 were routinely used. For passaging and seeding, MC3T3-E1 cells were removed from the flasks by treatment with 0.25% Trypsin-EDTA (Sigma, St. Louis, MO, USA). RAW 264.7 macrophages were passaged for three to five times after thawing before use in experiments, and passaged by gently scraped from the flasks using a sterile cell scraper (Sigma, St. Louis, MO, USA). Cells were counted using an automated cell counter (TC20, BioRad, Hercules, CA, USA) with trypan blue staining. prior to cell seeding on coated disks, on the side that had the biomolecules adsorbed. Prior to FGF-2 application and cell seeding, the coated disks were UV sterilized for 10 min on each side. After FGF-2 application, the disks were incubated in a α-MEM medium for 35 min in non-treated tissue culture 12-well plates (Corning Inc., Corning, NY, USA). MC3T3-E1s were then seeded at 4 × 10^4^ cells/cm^2^ while the RAW 264.7 cells were seeded at 3 × 10^4^ cells/cm^2^, and both were incubated at 37 °C and 5% CO_2_.

LIVE^®^ staining (Invitrogen Life Technologies, Grand Island, NY, USA) was performed, following the manufacturer’s protocol, as a terminal assay to measure the density of the living cells of both cell types. The assays were conducted primarily out to six days, as acute inflammation occurs during the first week of implantation, and previous studies had shown MC3T3-E1 cell access to the cytotoxic compound occurred on day three [21]. The earliest time point measured was 4 h, to allow for sufficient cell attachment. At each time point, the disks with cells were transferred to a new 12-well plate (Corning Inc., Corning, NY, USA) and washed with phosphate buffered saline solution (PBS) to remove non-adherent cells, and incubated at room temperature for 30 min in LIVE^®^ staining reagents. After 30 min of incubation, the disks were flipped over for imaging the side with the cells at 100× magnification, using an inverted microscope (TE300, Nikon Instruments, Melville, NY, USA) equipped with a camera (Spot 18.2 Color Mosaic, Diagnostic Instruments, Sterling Heights, MI, USA) and imaging software (Spot Imaging, Diagnostic Instruments, Sterling Heights, MI, USA). 

The cell density was quantified as the average percent fluorescent area of three of the threshold images per well with three replicates via ImageJ software (U.S. National Institutes of Health, Bethesda, MD, USA) as follows. The percent cell death was calculated by subtracting the average percent live stained area of the AntiA group, normalized by its respective AntiA-negative control from 100%. All of the experiments were repeated at least three times. The CellTiter-Blue^®^ (CTB) (Promega Corporation, Madison, WI, USA) cell viability assay was used on the bCaP only disks for comparison to the live staining method, and were found to be comparable (data are not shown here). 

#### 2.2.2. Evaluation of Cell Access Mechanism

Scanning electron microscopy (SEM) (JSM-5900LV, JEOL USA, Inc., Peabody, MA, USA) was used to characterize the thickness of the coatings and to identify the coating morphology changes after coating incubation in a culture medium with or without cells. The effects from both cell types were evaluated by SEM. The MC3T3-E1 or RAW 264.7 cells were cultured on disks coated with AntiA-bCaP-FGF2 or AntiA-bCaP-PEM30-FGF2. After LIVE^®^ staining was performed, the cells were removed by incubation in Trypsin-EDTA (Sigma, St. Louis, MO, USA) and were washed three times with ultrapure water (reverse osmosis), and dried with a series of ethanol solutions for SEM imaging. The disks with PEM coatings were critically point dried after ethanol dehydration (LEICA EM CPD030, Leica Microsystems Inc., Buffalo Grove, IL, USA) to preserve the delicate surface structure of the PEM film. For consistency, the bCaP only coated disks were also critically point dried. The disks were sputtered coated (DESK V, Denton Vacuum, LLC, Manchester, NJ, USA) and imaged using a TM-1000 SEM (Hitachi High-Technologies Corporation, Tokyo, Japan). The SEM analysis was conducted on five areas per quadrant, and on at least three samples per group.

### 2.3. Statistical Analysis

Statistical significances were determined using Graph Pad Prism software by unpaired t-tests if only two groups were in the study or by one-way analysis of variance (ANOVA) with Tukey post-tests for multiple comparisons in larger studies with *p* values >0.05 being considered statistically significant.

## 3. Results

### 3.1. Characterizations of bCaP-PEM Coating

#### 3.1.1. Changing bCaP Layer Thickness in the Presence of PEM

**Figure 2:**
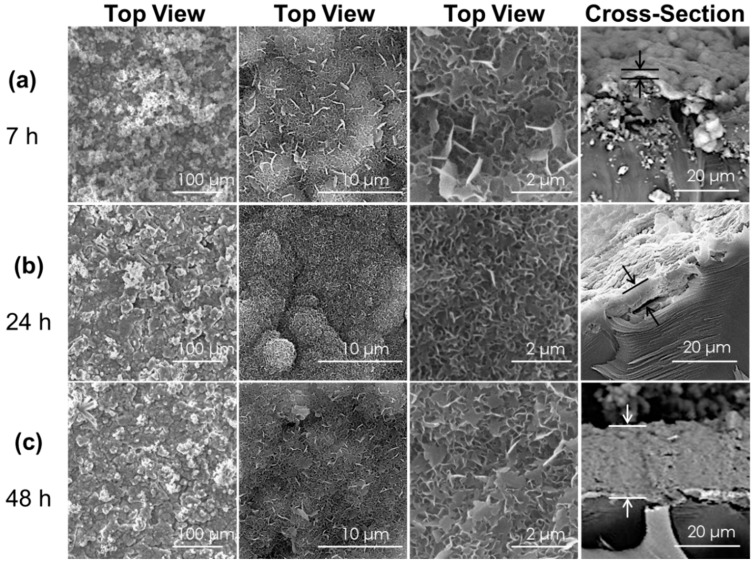
Scanning electron microscopy images of the top surface morphology and cross-section of bCaP coating before PEM and before cell culture. Times shown on right column are lengths of time in the simulated body fluid solution. (**a**) bCaP (7 h) thickness = 1.8 ± 0.7 μm; (**b**) bCaP (24 h) thickness = 5.8 ± 1.8 μm; and (**c**) bCaP (48 h) thickness = 24.0 ± 2.4 μm.

Studies were undertaken to determine if increasing the bCaP barrier layer thickness would increase the timing of cell access to the factor embedded below the bCaP barrier layer. Increasing the bCaP layer thickness was achieved by increasing the incubation time in the simulated body fluid solution. Thicknesses were measured from the SEM images of the side view of disks coated with bCaP(7 h), bCaP(24 h), and bCaP(48 h). The coating thicknesses were 1.8 ± 0.7 μm (7 h), 5.8 ± 1.8 μm (24 h), and 24.0 ± 2.4 μm (48 h) (Figure 2). 

**Figure 3:**
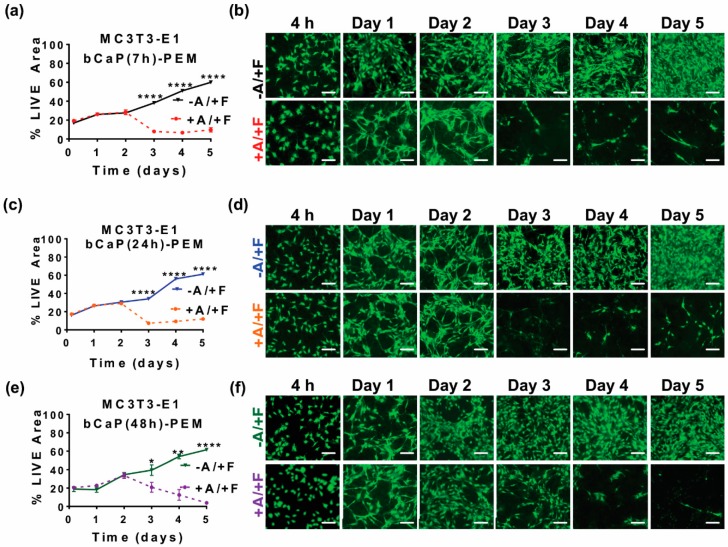
MC3T3-E1 osteoprogenitor cells cultured on bCaP of varying thickness with PEM present: (**a**) percent LIVE^®^ stained area of MC3T3-E1s cultured on bCaP(7 h)-PEM30-FGF2 without Antimycin A (−A/+F) and AntiA-bCaP(7 h)-PEM30-FGF2 with Antimycin-A (+A/+F) (**** *p* < 0.0001); (**b**) fluorescent LIVE^®^ stained images of MC3T3-E1 cells on bCaP(7 h)-PEM30-FGF2 (−A/+F) and AntiA-bCaP(7 h)-PEM30-FGF2 (+A/+F); (**c**) percent LIVE^®^ stained area of MC3T3-E1s cultured on bCaP(24 h)-PEM30-FGF2 (−A/+F) and AntiA-bCaP(24 h)-PEM30-FGF2 (+A/+F) (**** *p* < 0.0001); (**d**) fluorescent LIVE^®^ stained images of MC3T3-E1 osteoprogenitor cells on bCaP(24 h)-PEM30-FGF2 (−A/+F) and AntiA-bCaP(24 h)-PEM30-FGF2 (+A/+F); (**e**) percent LIVE^®^ stained area of MC3T3-E1 osteoprogenitor cells cultured on bCaP(48 h)-PEM30-FGF2 (−A/+F) and AntiA-bCaP(48 h)-PEM30-FGF2 (+A/+F) (* *p* < 0.05, ** *p* < 0.01, **** *p* < 0.0001); and (**f**) fluorescent LIVE^®^ stained images of MC3T3-E1 cells on bCaP(48 h)-PEM30-FGF2 (−A/+F) and AntiA-bCaP(48 h)-PEM30-FGF2 (+A/+F). Time points 4 h, one–five days. Scale bar = 100 μm.

Cell access to the cytotoxic AntiA embedded below bCaP was quantified by measuring the density of live cells remaining after cell culture directly on the coated disks. On day three, there was an abrupt onset of cell death, as evidenced by significant decreases in LIVE^®^ staining, indicating that AntiA was accessed by the MC3T3-E1 cells on day three of the cell culture on both the thinner AntiA-bCaP(7 h)-PEM30-FGF2 (Figure 3a,b) and the thicker AntiA-bCaP(24 h)-PEM30-FGF2 (Figure 3c,d). Depositing an even thicker bCaP layer by refreshing the SBF solution during the coating deposition slowed, but did not stop the access of MC3T3-E1 cells to AntiA on day 3 (Figure 3e,f). There was a minor, but significant difference, as compared to the complete access on day three by the cells cultured on both AntiA-bCaP(7 h)-PEM30-FGF2 and AntiA-bCaP(24 h)-PEM30-FGF2. 

#### 3.1.2. Increasing Number of PEM Bilayers

**Figure A1:**
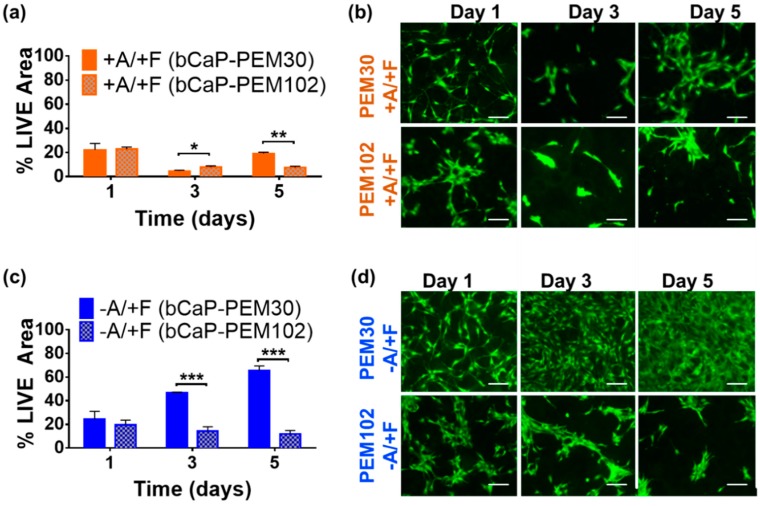
Increasing the number of PEM bilayers: (**a**) percent LIVE^®^ stained area of MC3T3-E1s cultured on AntiA-bCaP-PEM30-FGF2 as compared to cells cultured on AntiA-bCaP-PEM102-FGF2 (* *p* < 0.05, ** *p* < 0.01 and *** *p* < 0.001); (**b**) fluorescent LIVE^®^ stained images at one, three, and five days of culture on AntiA-bCaP-PEM30-FGF2 as compared to cells on AntiA-bCaP-PEM102-FGF2; (**c**) percent LIVE^®^ stained area of MC3T3-E1s cultured on control bCaP-PEM30-FGF2 as compared to cells on bCaP-PEM102-FGF2 (*** *p* < 0.01); and (**d**) fluorescent LIVE^®^ stained images at one, three, and five days of culture on bCaP-PEM30-FGF2, as compared to cells cultured on bCaP-PEM102-FGF2. Scale bar = 100 μm.

In attempts to change the timing of access to the factors by the cells, the PEM thickness and composition were varied. A thicker PEM film was expected to increase the time required by the cells to access the embedded AntiA, yet this was not observed. Increasing the number of PEM bilayers from 30 to 102 bilayers resulted in cell access to AntiA on day three, the same time as PEM 30, as measured by a significant decrease in the LIVE^®^ staining of MC3T3-E1s cultured on AntiA-bCaP-PEM102-FGF2 on day three (Figure A1a,b). However, in studies without any AntiA, decreased cell viability was observed when the PEM thickness was increased to 102, which continued out to day five, as compared to the viability on the original bCaP-PEM30-FGF2 (Figure A1c,d). Thus, increasing the PEM layer number to 102 led to deleterious effects on cell viability and PEM102 was not selected for further studies with macrophages.

#### 3.1.3. Use of D-Enantiomers: PEM-D

**Figure A2:**
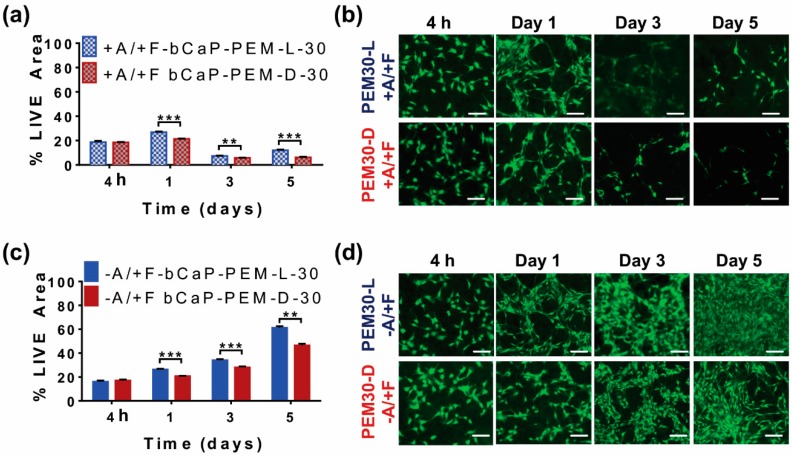
Effects of varying polyamino acid enantiomer type. (**a**) percent LIVE^®^ stained area of MC3T3-E1s cultured on AntiA-bCaP-PEM30-D-FGF2 (orange) as compared to cells cultured on AntiA-bCaP-PEM30-L-FGF2 (Green) (** *p* = 0.003, *** *p* <0.0004); (**b**) fluorescent LIVE^®^ stained images at 4 h, and one, three, and five days of culture on AntiA bCaP-PEM30-L-FGF2 as compared to cells on AntiA-bCaP-PEM30-D-FGF2; (**c**) percent LIVE^®^ stained area of MC3T3-E1s cultured on bCaP-PEM30-D-FGF2 (red) as compared to cells cultured on bCaP-PEM30-L-FGF2 (blue), (** *p* = 0.003, *** *p* < 0.0004); and (**d**) fluorescent LIVE^®^ stained images at 4 h, 1 and one, three, and five days of culture on bCaP-PEM30-D-FGF2 as compared to cells cultured on bCaP-PEM30-L-FGF2. Scale bar = 100 μm.

Another attempt to alter the timing of the factor release was made by using the d enantiomer form of the polyamino acids, compared to the l-form in the original coatings, because it had been previously shown that the d-form could delay cell access to factors over the short term (e.g., 12 h or less) [43]. The PEM-D coatings resulted in MC3T3-E1 cells accessing AntiA on the same time point (day three) as the coatings made with PEM-L (Figure A2a,b). With the FGF-2 adsorbed in the outer layer, the cells proliferated more rapidly on the bCaP/PEM coatings made with the l-form, compared with the d-form (Figure A2c,d). The PEM-D thus had a negative effect on the cell viability, as seen from a significantly decreased percentage of LIVE^®^ staining of MC3T3-E1s cultured on bCaP-PEM30-l-FGF2 on days one, three, and five, as compared to the cells on the bCaP-PEM30-l-FGF2 disks. Given that the cell viability was adversely affected by using d enantiomers compared to l-form, and thicker layers of the PEM30-D were not investigated further and d enantiomers were not used in the macrophage cell bioassays.

### 3.2. Characterization of the Coatings bCaP without PEM

#### 3.2.1. Effects of Removing PEM Film

**Figure 4:**
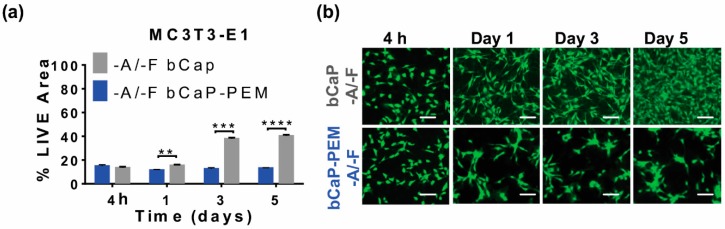
MC3T3-E1 osteoprogenitor cells cultured on bCaP coating or bCaP-PEM without any factors to examine the biocompatibility of the biomaterial coatings. (**a**) Percent of LIVE^®^ stained area of MC3T3-E1s on bCaP (24 h) (−A/−F) vs. bCaP (24 h)-PEM (−A/−F), and (**b**) fluorescent LIVE^®^ stained images of MC3T3-E1 cells on bCaP (24 h) and bCaP (24 h)-PEM. Scale bar = 100 μm.

The effect of the PEM film on the kinetics of cell access to factors embedded under the bCaP-PEM system was tested by depositing a bCaP coating only with or without AntiA below and leaving off the overlying PEM film. The cells reacted to being cultured on the bCaP alone, without PEM and without FGF-2 with enhanced cell proliferation over time, as compared to bCaP/PEM without FGF-2. In the coatings without any AntiA, a significant increase in the proliferation, as indicated by percent LIVE^®^ stained area of MC3T3-E1 cells, was observed when the cells were cultured on bCaP-only (grey), as compared to the cells cultured on bCaP-PEM (blue) at day one, three, and five (Figure 4a,b).

**Figure 5:**
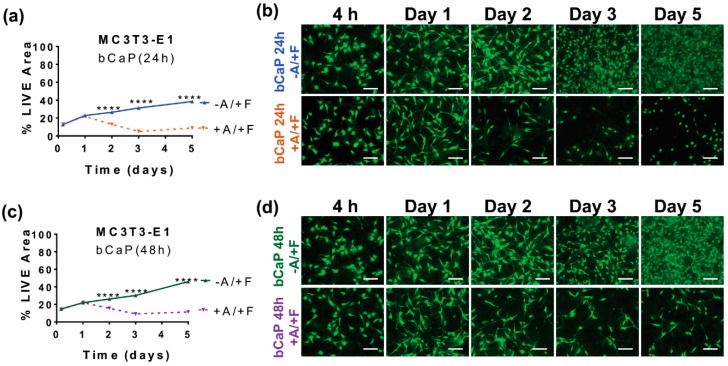
MC3T3-E1 osteoprogenitor cells cultured on bCaP of varying thickness without PEM: (**a**) percent LIVE^®^ stained area of MC3T3-E1s on bCaP(24 h)-FGF2 (−A/+F) vs. AntiA-bCaP(24 h)-FGF2 (+A/+F); (**b**) fluorescent image of LIVE^®^ stain of MC3T3-E1s on bCaP(24 h)-FGF2 (−A/+F) and on AntiA-bCaP(24 h)-FGF2 (+A/+F); (**c**) percent LIVE^®^ stained area of MC3T3-E1s on bCaP(48 h)-FGF2 (−A/+F) vs. AntiA-bCaP(48 h)-FGF2 (+A/+F); and (**d**) fluorescent image of LIVE^®^ stain of MC3T3-E1s on bCaP(48 h)-FGF2 (−A/+F) and on AntiA-bCaP(48 h)-FGF2 (+A/+F), (**** *p* < 0.001). Scale bar = 100 μm.

In other experiments without a PEM film, the MC3T3-E1 cells were either cultured on a bCaP(24 h) or bCaP (48 h) coating with AntiA embedded beneath the bCaP coating, and with FGF2 adsorbed on top of the bCaP coating. On the bCaP(24 h) coating without PEM, the AntiA was accessed by the MC3T3-E1 cells a day earlier, on day two, with less cell viability over the remaining time points studied (Figure 5a,b) compared to the bCaP-PEM coating. With PEM, the MC3T3-E1s on AntiA-bCaP-PEM30-FGF2 initially proliferated and then abruptly accessed the embedded AntiA on day 3 and then showed a slight recovery. Depositing a thicker bCaP(48 h) layer without PEM resulted in a more gradual access to the cytotoxic AntiA than bCaP(24 h), but cell access was still observed on day 2 as measured by reduced cell viability (Figure 5c,d).

#### 3.2.2. Pulsatile Delivery from bCaP

**Figure 6:**
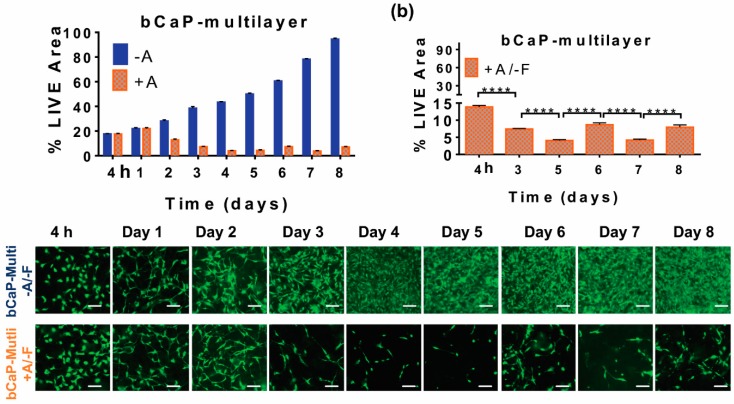
Kinetics of cell access when MC3T3-E1 osteoprogenitors were cultured on multilayers of bCaP without PEM: (**a**) percent LIVE^®^ stained area of MC3T3-E1s cultured on bCaP-1-bCaP2 (blue) as compared to cells cultured on AntiA-bCaP1-AntiA-bCaP2 (orange); (**b**) percent LIVE^®^ stained area of MC3T3-E1s cultured on AntiA-bCaP1-AntiA-bCaP2 (orange); (**c**) fluorescent images of cells on days 2, 3, 5, 6, 7 and 8. (**** *p* < 0.001) Scale bar = 100 μm.

In an attempt to achieve pulsatile delivery from the bCaP delivery system, a double layer of AntiA-bCaP with two separate AntiA doses applied below each bCaP layer, was tested in the cell culture bioassays. Based on the extended decreases in live staining, AntiA was gradually accessed by MC3T3-E1 cells starting on day two, and the access continued until day five (Figure 6a). The LIVE^®^ staining showed that the cell viability increased on day six, until the second dose of AntiA was abruptly accessed on day seven, followed by cell regrowth a day later, on day eight (Figure 6). There was no significant difference in the LIVE^®^ staining between the lows on day five and seven for the AntiA group, indicating maximum effects on those two days, with a comparable regrowth initiating afterwards. No significant differences in viability were noticed on day six and eight, indicating that the cell recovery occurred to the same extent at these two time points.

### 3.3. Studying Macrophages to Mimic Cells Present during Inflammation and Bone Remodeling

**Figure 7:**
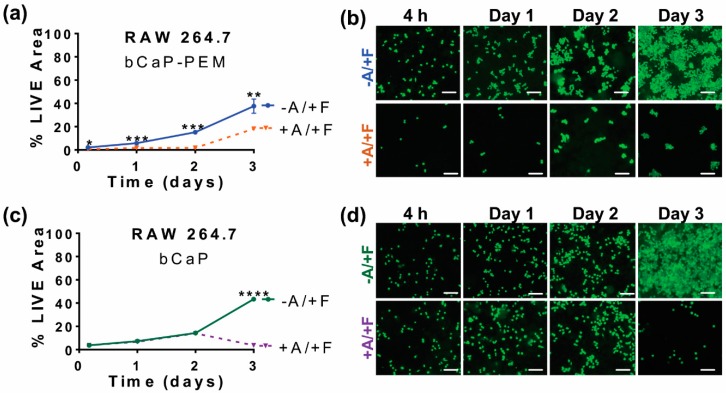
RAW 264.7 macrophages cultured on bCaP or bCaP-PEM: (**a**) percent LIVE^®^ stained area of RAW 264.7 cells cultured on bCaP-PEM30-FGF2 (−A/+F) as compared to cells cultured on AntiA-bCaP-PEM30-FGF2 (+A/+F) (* p ≤ 0.05, ** *p* ≤ 0.01, *** *p* ≤ 0.001); (**b**) fluorescent LIVE^®^ stained images of RAW 264.6 cells cultured on bCaP-PEM30-FGF2 (−A/+F) as compared to cells cultured on AntiA-bCaP-PEM30-FGF2 (+A/+F) at 4 h, 1, 2, and 3 days of culture; (**c**) percent LIVE^®^ stained area of RAW cells on bCaP coating (**** *p* < 0.001); and (**d**) fluorescent LIVE^®^ stained images of cells cultured on bCaP-FGF2 (−A/+F) as compared to cells cultured on AntiA-bCaP- FGF2 (+A/+F) at 4 h, and 1–3 days of culture. Scale bar = 100 μm.

Studies with RAW 264.7 mouse macrophages, in addition to those with MC3T3-E1 mouse calvarial osteoprogenitors, were conducted so as to understand the delivery profile that might occur during early inflammation when macrophages are present. Changing the cell type to macrophages altered the cell access kinetics to the AntiA. The RAW 264.7 cells immediately accessed the embedded AntiA on the disks coated with AntiA-bCaP-PEM30-FGF2, observed by the significant decrease in LIVE^®^ at the first time point, 4 h, and throughout the duration of the study (Figure 7a,b). This was in contrast to the MC3T3-E1 cells, which accessed AntiA on day three of the culture on this coating (Figure 2c,d). Removing the PEM film slowed the access of the RAW 264.7 macrophages, resulting in access on day three to the embedded AntiA (Figure 7c,d).

### 3.4. Cells Access Mechanism as Measured by SEM Imaging

**Figure 8:**
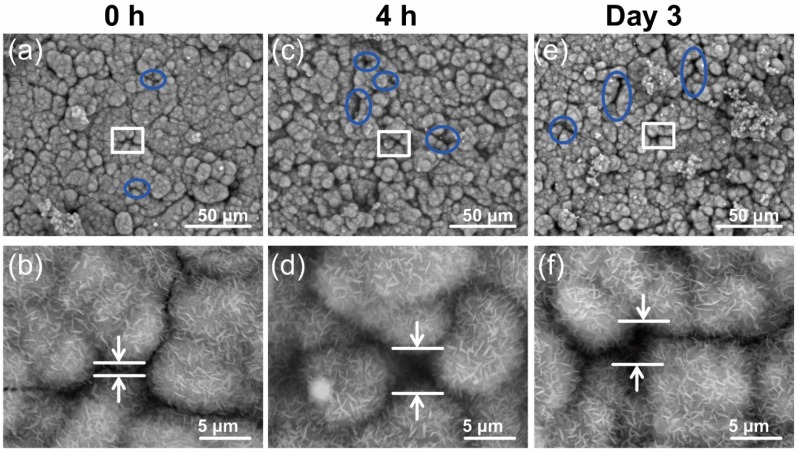
SEM imaging of (+A/+F)-bCaP(24 h) coated disk incubated in media with no cells: (**a**,**b**) AntiA-bCaP(24 h)-FGF2 before incubation in culture medium; (**c**,**d**) low and high magnification of +A/+F AntiA-bCaP(24 h)-FGF2 incubated in culture medium for 4 h; and (**e**,**f**) low and high magnification of +A/+F AntiA-bCaP(24 h)-FGF2 incubated in culture medium for three days. The white squares indicates the areas shown at higher magnification in the row below. Blue circles highlight the openings between crystal junctions that widened with time in culture medium.

SEM imaging was used before and after the cell culture on the disks coated with bCaP (24 h) or bCaP (24 h)-PEM, to investigate the changes in the coating integrity resulting from contact with both of the cell types during their culture time on the disk. SEM revealed that the incubation of the bCaP (24 h) coated disk in the culture medium alone, without cells, resulted in changes in the surface morphology. A dissolution process occurred, which opened the junctions between the islands of bCaP (24 h) crystals (wider cracks were noticed on both 4 h and day three) (Figure 8). As the medium incubation caused wider cracks between the crystal clusters of the bCaP (24 h) coating, it is speculated that that is why the MC3T3-E1 cells were able to penetrate, using their cell processes, through the widening cracks, to directly access AntiA on day two. The AntiA release from the coating without cells present on day two had been shown to be negligible in our previous studies, based on the lack of toxicity observed when adding release media collected on day two to cell cultures [21]. 

**Figure 9:**
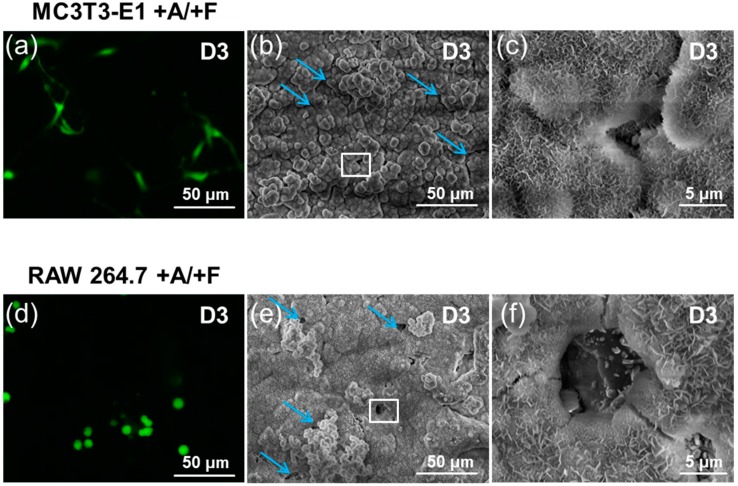
SEM imaging of (+A/+F)-bCaP(24 h) with either MC3T3-E1 or RAW 264.7 cells: (**a**) day3 LIVE^®^ staining of MC3T3-E1 on +A/+F AntiA-bCaP(24 h)-FGF2; (**b**,**c**) low and high magnification SEM of AntiA-bCaP(24 h)-FGF2 after three days of MC3T3-E1 culture; (**d**) day three LIVE^®^ staining of RAW 264.7 cells on AntiA-bCaP(24 h)-FGF2; and (**e**,**f**) AntiA-bCaP(24 h)-FGF2 after three days of RAW 264.7 culture. The white squares indicates the areas shown at higher magnification to the right of the image. Blue arrows point to other openings in the bCaP coatings similar to those in the higher magnification image outlined in the white box.

In addition to widening the junctions between the crystal islands, SEM revealed that the RAW cells dissolved bCaP (24 h) to create micro-openings in the AntiA-bCaP (24 h)-FGF-2 coating on day three of the culture (Figure 9). These holes would allow them to get access to the embedded AntiA on the bCaP (24 h) coating, and the timing coincided with the loss of the cell viability observed in the studies. The average number of micro-openings created by the macrophages on day three of the culture, as counted manually, was 25 ± 9.5 per disk.

**Figure A3:**
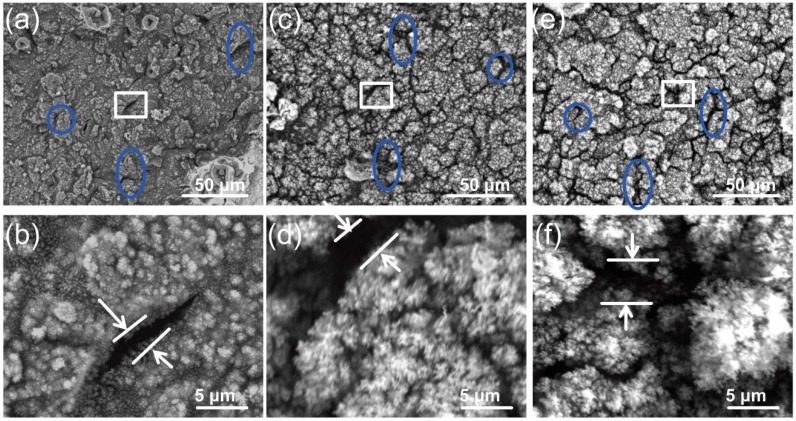
SEM imaging of (+A/+F)-bCaP(24 h)-PEM before and after incubation in media only, no cells. (**a**,**b**) AntiA-bCaP(24 h)-PEM-FGF2 before incubation in culture medium (0 h); (**c**,**d**) low and high magnification of +A/+F AntiA-bCaP(24 h)-PEM-FGF2 incubated in culture medium for 4 h; and (**e**,**f**) low and high magnification of +A/+F AntiA-bCaP(24 h)-PEM-FGF2 incubated in culture medium for three days. Blue circles in (**a**,**c** and **e**) highlight other openings between crystal junctions that are similar to those selected within the white boxes for higher magnification in the (**b**,**d** and **f**).

**Figure A4:**
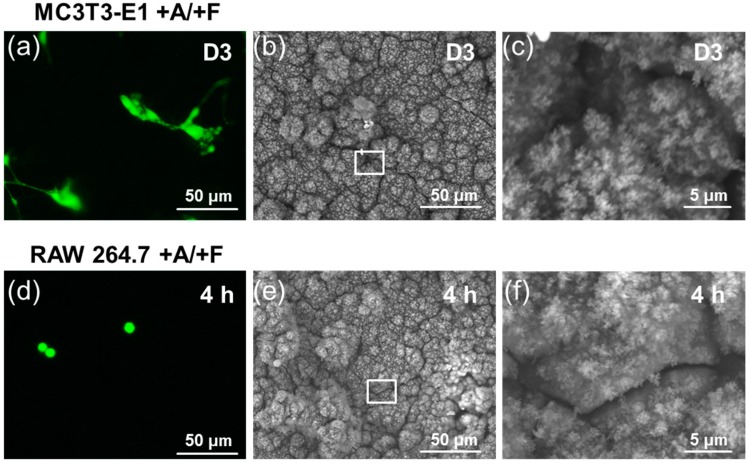
SEM imaging of (+A/+F)-bCaP(24 h)-PEM) with either MC3T3-E1 or RAW 264.7 cells: (**a**) day three LIVE^®^ staining of MC3T3-E1 on +A/+F AntiA-bCaP(24 h)-PEM-FGF2; (**b**,**c**) low and high magnification SEM of AntiA-bCaP(24 h)-PEM-FGF2 after three days of MC3T3-E1 culture; white square in (**b**) shows area of higher magnification in (**c**); (**d**) 4 h LIVE^®^ staining of RAW 264.7 cells on AntiA-bCaP(24 h)-PEM-FGF2; and (**e**,**f**) AntiA-bCaP(24 h)-FGF2 after 4 h of RAW 264.7 culture. White square in (**e**) shows area of higher magnification shown in (**f**).

SEM revealed that the incubation of bCaP with a PEM film in the culture medium alone without cells also resulted in changes in the surface morphology. The cracks widened after 4 h and three days incubation time (Figure A3). The cracks were slightly narrower and sharper after the MC3T3-E1 osteoprogenitors were cultured on bCaP-PEM coated disks than when the RAW 264.7 macrophages were cultured on bCaP-PEM coated disks (representative images shown in Figure A4). The RAW 264.7 cells died immediately upon contact with the AntiA-bCaP (24 h)-PEM coated disks, and the dead cells did not create micro-openings in AntiA-bCaP (24 h)-PEM on day three of the culture.

## 4. Discussion

In this study, the relationship between the thickness of the components of the bCaP/PEM delivery system and the step-wise biological response of the osteoprogenitor cells and the macrophage cells to the system were investigated. The PEM coating thickness can be highly controlled during the polyelectrolyte multilayer deposition on both the two-dimensional (2D) or 3D substrates by adjusting the number of times the samples are immersed in the polyelectrolyte solutions; however, this did not have a large effect. There was an unexpected loss in the cell viability from increasing the PLGlut/PLLys PEM bilayers from 30 to 102. The reduced cell viability with bCaP-PEM, as compared to bCaP alone, was attributed to the presence of PLLys within the coating. The PLLys is known to have a slightly cytotoxic effect with time, and a concentration-dependent effect has been observed in an MTT cytotoxicity assay [44,45]. 

The bCaP barrier layer thickness was also altered in an attempt to change the kinetics of the cell access to the factors embedded under either bCaP-PEM or bCaP, without the PEM film. Increasing or decreasing the incubation time in the concentrated SBF solutions resulted in a corresponding increase or decrease in the bCaP coating thickness, which was anticipated based on the related results of the bCaP growth on titanium [38,39,40]. Surprisingly, the much thicker coating only slightly delayed the MC3T3-E1 osteoprogenitor access to the cytotoxic AntiA below. Based on the SEM analysis and the literature, this is likely because the bCaP layer has a nano-porous structure due to the nucleation process, which results in islands of crystalline mineral with pores at the junctions [46,47,48]. The cracks that are incurred during the ethanol dehydration step prior to cell seeding also govern the cell access rate. Pore reduction or increasing crystallinity through heat treatment or an alternate deposition process, like plasma spray, would likely entomb the factor completely or inactivate it, preventing biological interactions for a week or more, which is undesirable for bone defect healing. As an alternate approach, there are many modifications to the PEM layer that could be made, such as substituting the PLLys and PLGlut polyamino acids with other molecules, such as chitosan or hyaluronan, that could be investigated in future studies to change the kinetics of cell access.

The largest differences in the structure–function relationships of the bCaP/PEM coatings were seen when the cell type being used for the drug access studies was changed from osteoprogenitors to macrophages. Macrophages are one of the very first cells interacting with implanted materials [49] and have an essential role in regulating bone remodeling, tissue repair, and tissue response to biomaterials [3,50,51]. Macrophages are known to have the ability to quickly degrade or phagocytose materials. For example, the RAW 264.7 murine macrophage cell line has the ability to degrade non-cross-linked polyelectrolyte multilayer films [52]. In this study, RAW 264.7 cells showed an immediate, without delay, access to the embedded AntiA when cultured on bCaP-PEM, but a longer two day delay when cultured on bCaP without PEM. This counterintuitive, enhanced access of macrophages after removing a PEM film is likely associated with changes in the bCaP structure, which occur during application of PEM. Previous studies showed that the crystallinity of the bCaP coating was reduced when a PEM film was applied to bCaP [21]. This may explain the enhanced and expedited degradation process of bCaP/PEM by the RAW 264.7 cells. 

To better assess the differences in the mechanism of cell access to the embedded AntiA by the osteoprogenitors versus the macrophages, SEM imaging was performed to study the morphological changes to the coated disks before and after cell culture. As it was found that the incubation of bCaP or bCaP-PEM coated disks in a culture medium opened the cracks between the crystal island junctions, and knowing that MC3T3-E1 cells are not capable of the resorption of calcium phosphate, it is believed that MC3T3-E1 was able to access the embedded factor by extending the cell processes through the cracks that widened over time. The RAW 264.7 cells had a different interaction with the coating, and the cell created pits in the bCaP coatings were seen on day three of the cell culture on AntiA-bCaP corresponding with the sudden cell death on day three. Macrophages and other phagocytic cells are known to have an ability to reduce the micro-environmental pH that would dissolve carbonated hydroxyapatite based coating [53]. The cell-based degradation of the biomaterials delivery system, particularly the biomimetic materials that are easily degraded by cells, can be exploited to achieve the desired release rate.

At the molecular level, bone healing is driven by three main factors, pro-inflammatory factors, coupled osteoblast-osteoclast activity, and angiogenic factors. The understanding of the cross-talk between the inflammatory cells, such as macrophages and osteoprogenitors, is expanding [1,2,54]. As the importance of the interactions between the immune and skeletal systems is recognized, it has become clear that macrophages are novel therapeutic targets [55]. Delivery systems that can modulate macrophage transitions during healing provide a new means of improving bone healing. The bCaP system is ideally suited to impact both osteoprogenitors and macrophages, given the long standing recognition of the osteopromotive effects of calcium phosphates on bone formation, and these effects on macrophages seen in the present studies during the first three days of culture, which would apply to modulating inflammation. 

## 5. Conclusions

These studies show that osteoprogenitor cell access rates to a cytotoxic compound embedded below a bone-like apatite coating (bCaP) were difficult to alter, despite increasing the bCaP coating thickness five times, from 5.8 ± 1.8 μm to 24.0 ± 2.4 μm. Varying the PEM coating thickness or subtle changes to the chemical structure of the polyelectrolytes (l- to d-enantiomers) also did not alter the delivery kinetics, and in fact reduced the biocompatibility of the PEM film. By removing the PEM coating from the bCaP-PEM system, macrophages, but not osteoprogenitors, had a delayed access, rather than immediate, due to the modification of the bCaP layer during PEM application. A subtle pulsatile delivery was accomplished by depositing two layers of bCaP with AntiA applied below each layer. The varying cell type profoundly affected the kinetics of delivery due to (i) the enhanced ability of macrophages over osteoprogenitors to degrade the bCaP only coating, and (ii) reduced the bCaP crystallinity due to the PEM coating procedure, which caused expedited access to the second factor by the macrophage cells, but not the larger osteoprogenitors that relied on cracks in the coating to access the embedded factor. These studies demonstrate how cellular interactions are sensitive to the nanostructure of the biomaterial, and emphasize the need for testing multiple cell types in order to understand cell-biomaterial interactions that will occur upon in vivo implantation.
